# Effects of the Heterointerface on the Growth Characteristics of a Brownmillerite SrFeO_2.5_ Thin Film Grown on SrRuO_3_ and SrTiO_3_ Perovskites

**DOI:** 10.1038/s41598-020-60772-2

**Published:** 2020-03-02

**Authors:** Janghyun Jo, Venkata Raveendra Nallagatlla, Susant Kumar Acharya, Youngho Kang, Yoonkoo Kim, Sangmoon Yoon, Sangmin Lee, Hionsuck Baik, Seungwu Han, Miyoung Kim, Chang Uk Jung

**Affiliations:** 1 Department of Materials Science and Engineering and Research Institute of Advanced Materials, Seoul National University Seoul, 08826 Republic of Korea; 2 Department of Physics and Oxide Research Centre, Hankuk University of Foreign Studies, Yongin, 17035 Republic of Korea; 3 Seoul Center, Korea Basic Science Institute, Seoul, 136-713 Republic of Korea

**Keywords:** Engineering, Materials science, Nanoscience and technology

## Abstract

Manipulation of the heterointerfacial structure and/or chemistry of transition metal oxides is of great interest for the development of novel properties. However, few studies have focused on heterointerfacial effects on the growth characteristics of oxide thin films, although such interfacial engineering is crucial to determine the growth dynamics and physical properties of oxide heterostructures. Herein, we show that heterointerfacial effects play key roles in determining the growth process of oxide thin films by overcoming the simple epitaxial strain energy. Brownmillerite (SrFeO_2.5_; BM-SFO) thin films are epitaxially grown along the *b*-axis on both SrTiO_3_(001) and SrRuO_3_/SrTiO_3_(001) substrates, whereas growth along the *a*-axis is expected from conventional epitaxial strain effects originating from lattice mismatch with the substrates. Scanning transmission electron microscopy measurements and first principles calculations reveal that these peculiar growth characteristics of BM-SFO thin films originate from the heterointerfacial effects governed by their distinct interfacial structures. These include *octahedral connectivity* between dissimilar oxides containing different chemical species and a *peculiar transition layer* for BM-SFO/SrRuO_3_/SrTiO_3_(001) and BM-SFO/SrTiO_3_(001) heterostructures, respectively. These effects enable subtle control of the growth process of oxide thin films and could facilitate the fabrication of novel functional devices.

## Introduction

The diverse properties and broad utility of transition metal oxides having the ABO_3_ perovskite structure and related structures result from strong coupling among the lattice, charge, spin, and orbital degrees of freedom. These basically originate from the strong hybridization between transition metal *d* and oxygen 2*p* orbitals^1,2^. Engineering such complex interactions has been recognized as a way to tailor the functional properties of transition metal oxides. In recent years, manipulating the heterointerface of transition metal oxides has enabled the development of novel phenomena such as two-dimensional free electron gases^3–7^, interfacial charge transfer^8,9^, high-*T*c superconductors^10^, and colossal magnetoresistance^11^; these have never been available in bulk equilibrium phases.

These new phenomena emerged through controlling the interface, which essentially modify the degree of orbital hybridization^12–14^. Such control is mediated by, for example, structural distortions, crystal symmetry, and oxygen coordination environments of oxide-based heterointerfaces. The interfacial structure and/or chemistry can also profoundly influence the growth dynamics of oxide thin films on foreign substrates. The resulting growth behaviours of oxide thin films would significantly affect the microstructures and physical properties of the thin films. Therefore, accurate determination of the oxide heterointerfacial structure is essential to understand the growth characteristics of oxide thin films and fabricate functional devices with desired physical properties.

Brownmillerite oxides such as SrFeO_2.5_ (BM-SFO) are of particular interest due to their wide range of physical properties including thermoelectricity, fast oxygen-ion transport, catalysis, and topotactic phase transformation at low temperatures^15,16^. These properties allow BM oxides to be exploited for extensive applications such as in solid-oxide fuel cells, sensors, membranes for oxygen separation, photon catalysis, and memristors^17–19^. BM oxides have a *perovskite-derived* structure with parallel rows of ordered oxygen vacancies that create tetrahedral chains directed along [101]_pc_ in every second (010)_pc_ octahedral plane. This results in three-dimensional A_2_B_2_O_5_ structures composed of alternate stacking of BO_6_ octahedral layers and sheets of BO_4_ tetrahedral layers^16^. Herein, the subscript ‘pc’ represents the perovskite unit cell in pseudocubic notation, which is derived from the intrinsic crystal structure of the BM phase^16^.

Controlling the direction of the oxygen vacancy channels in the BM structure, *i.e*., the growth orientation of BM oxides, has recently become important in a wide range of scientific and industrial areas. The oxygen vacancy channels are believed to form a pathway for easy and rapid oxygen-ion transport and to contribute to the ionic conductivity of the compound in applications such as solid-oxide fuel cells and resistive-switching memory devices^20–22^. The highly anisotropic structure originating from the ordered oxygen vacancy channels of the BM structure could alter the growth behaviour^21,23^. Many studies have exploited epitaxial strain to manipulate the growth orientation of BM thin films. They have demonstrated that oxygen-deficient channels of BM oxides can be arranged parallel or perpendicular to the substrate, depending on the interfacial strain resulting from lattice mismatch with the substrate^20,24^. In addition to controlling the growth process of the thin films by imposing epitaxial strain alone, various interfacial effects can extend the range of choice for engineering microstructures as well as the physical properties. However, these effects have been rarely reported, despite their great potential for manipulating the growth dynamics and consequential physical properties of oxide thin films.

Herein, we report novel interfacial effects that have a significant influence on the growth behaviour of oxide thin films by overcoming the conventional epitaxial strain effects. We investigated the microstructures of BM-SFO thin films grown on SrTiO_3_(STO)(001) and SrRuO_3_(SRO)/STO(001) substrates using transmission electron microscopy (TEM) and first principles calculations. We chose BM-SFO as a model system to examine the role of the interface on the growth dynamics of a complex oxide thin film. The BM-SFO thin films grew along *b*-axis on both the SRO/STO(001) and STO(001) substrates, while the films are predicted to grow along *a*-axis by the simple epitaxial strain effect. Even though the two substrates have the same in-plane lattice parameters, the thin films grown on both substrates displayed very different heterointerfacial structures and growth processes, suggesting additional key factors that control the growth behaviour of the thin films. We found that these differences in the growth processes of the thin films are attributed to interfacial effects determined by the heterointerfacial structures such as the *peculiar transition layer*, the different chemical species, and *octahedral connectivity*.

## Results and Discussion

**Figure 1:**
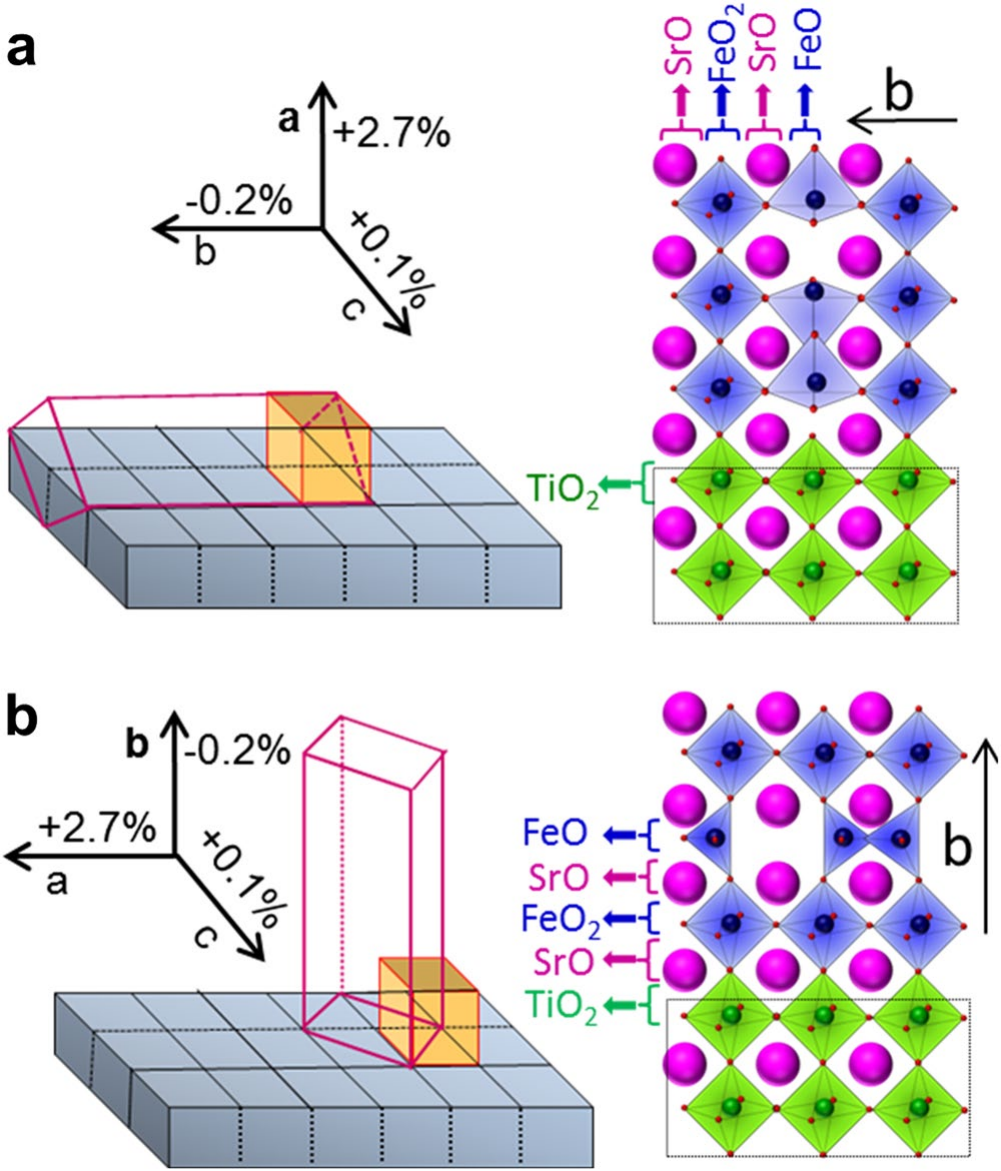
Schematic representations of the probable growth directions of BM-SFO thin films on STO and SRO substrates along the **(a)** *a*-axis and **(b)** *b*-axis. The orthorhombic unit cell of BM-SFO and its pseudocubic counterpart are illustrated by a transparent rectangular parallelepiped and a yellow cube, respectively. Lattice mismatch of BM-SFO with respect to STO at the interface is indicated along each coordinate direction in terms of the pseudocubic lattice parameters of BM-SFO. Atomic configurations at the interface are illustrated at the right side for both cases.

When studying the growth behaviour of metal oxide thin film, it is quite general to consider the epitaxial strain effect first and track down possible factors in detail. This procedure is also applicable to BM thin film despite its complicated crystal structure. Two lattice parameters of orthorhombic BM-SFO, *i.e*., *b*_*pc*_ = *b*/4 = 3.900 Å and ${c}_{pc}=c/\sqrt{2}=3.910\AA$, are very similar to that of cubic STO, *i.e*., *a* = 3.905 Å, while the lattice parameter of ${a}_{pc}=a/\sqrt{2}=4.010\AA$ of BM-SFO in the *a*-axis direction shows a relatively large lattice mismatch of about 2.7%. The nominal lattice mismatches of BM-SFO in pseudocubic notation with respect to the cubic STO substrate are +2.7%, −0.19%, and +0.08% along the *a*-, *b*-, and *c*-axes, respectively (Fig. 1). The in-plane area mismatch is also an important parameter for determining the growth characteristics of thin films because the interface is defined by two lattice vectors. Considering the lower surface area mismatch of −0.11% between the BM-SFO *bc*-plane and the STO(001) plane, *a*-axis growth of BM-SFO on the STO(001) substrate should be energetically much more favourable than *b*-axis growth, which has a larger surface area mismatch of +2.8%.

The SRO buffer layer has an orthorhombic crystal structure, but the distortion from the cubic structure is very small. Furthermore, the lattice parameter of the pseudocubic unit cell for bulk unstrained SRO (*a* = 3.923 Å) is similar to that of STO, thereby enabling perfectly coherent growth of an epitaxial SRO thin film on the STO(001) substrate to a thickness of about 100 nm. This suggests that the in-plane lattice parameters of the SRO layer are fixed to those of the STO(001) substrate. Thus, when considering only the epitaxial strain effect by lattice mismatch, BM-SFO thin films on both STO(001) and SRO/STO(001) substrates are expected to exhibit the same *a*-axis oriented growth behaviour.

**Figure 2:**
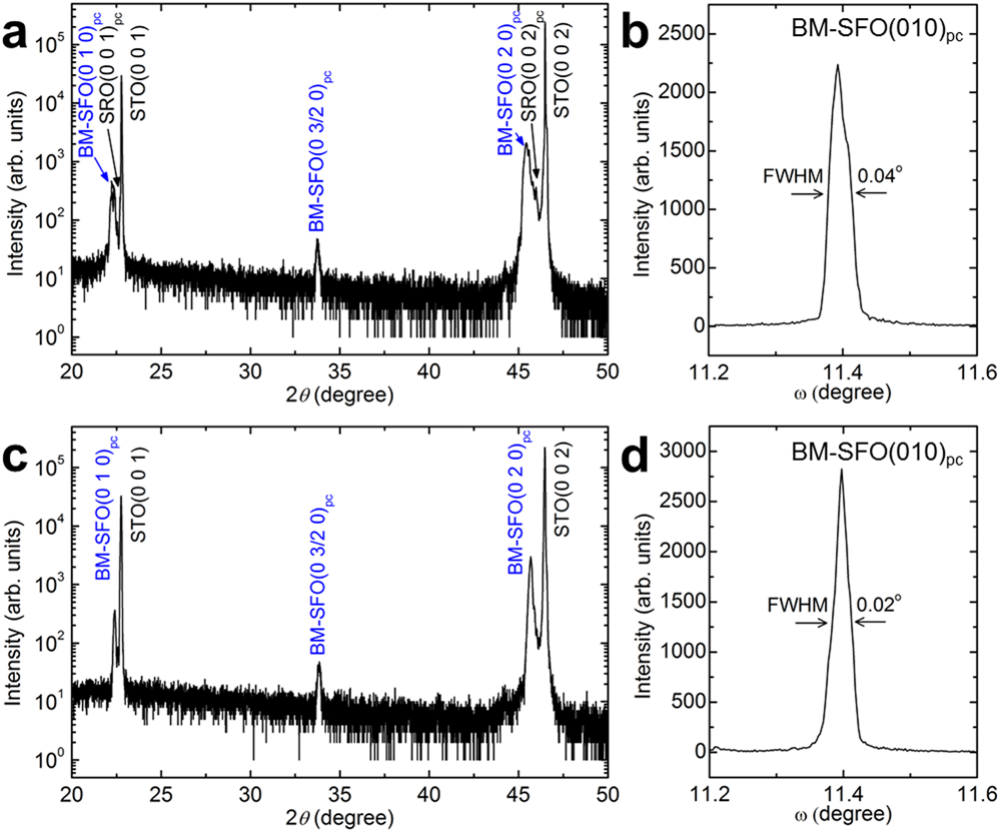
Structural characterization of BM-SFO thin films grown on SRO/STO(001) and STO(001) substrates using XRD. XRD *θ*−2*θ* scans of **(a)** BM-SFO/SRO/STO(001) and **(c)** BM-SFO/STO(001). XRD rocking curves of the BM-SFO(010)_pc_ peaks for **(b)** BM-SFO/SRO/STO(001) and **(d)** BM-SFO/STO(001). The FWHM of the rocking curve is indicated in each figure.

We performed X-ray diffraction (XRD) measurements to confirm the growth orientations of BM-SFO thin films on STO(001) and SRO/STO(001) substrates. Unexpectedly, the BM-SFO thin films revealed the *b*-axis oriented growth behaviour on both substrates rather than the *a*-axis oriented growth behaviour predicted by the epitaxial strain effect. Figure 2a presents an XRD *θ*−2*θ* scan of BM-SFO/SRO/STO(001). The pattern shows (010)_pc_ and (020)_pc_ diffraction peaks of BM-SFO near the respective SRO(001)_pc_ and (002)_pc_ peaks. The XRD rocking curve (Fig. 2b) shows a narrow full-width at half-maximum (FWHM) of 0.04°, which suggests good crystallinity of the thin film. Additionally, two-fold superstructure peaks corresponding to (0 1/2 0)_pc_ and (0 3/2 0)_pc_, caused by the ordering of oxygen vacancies, were observed in the XRD pattern. The XRD *θ*−2*θ* pattern of SFO/STO(001) also shows the (010)_pc_ and (020)_pc_ diffraction peaks of SFO near the STO(001)_pc_ and (002)_pc_ peaks, and the two-fold superstructure peaks (Fig. 2c). The XRD rocking curve shows a narrow FWHM of 0.02°, which suggests good crystallinity of the thin film (Fig. 2d). The appearance of the (0 *l*/2 0)_pc_ peaks in these XRD *θ*−2*θ* patterns clearly indicates the presence of the BM-SFO phase and its *b*-axis oriented growth on both SRO/STO(001) and STO(001) substrates^25^.

**Figure 3:**
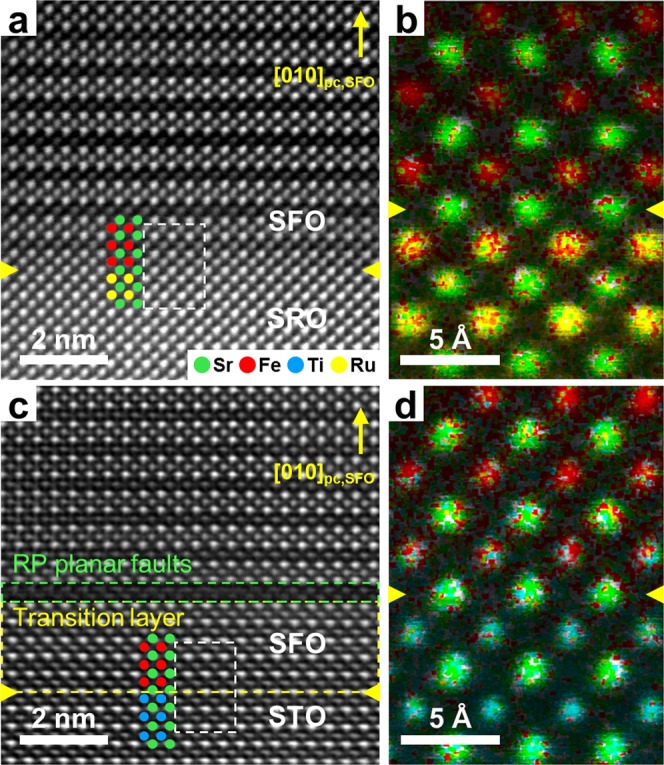
Atomic configurations of BM-SFO thin films at the interface. **(a,c)** Cross-sectional HAADF-STEM images of (**a**) BM-SFO/SRO/STO(001) and (**c**) BM-SFO/STO(001) at the interfaces. **(b,d)** Atomic-resolution EDS elemental maps at the interfaces of the regions marked by white dashed rectangles in (**a**) and (**c**), respectively. The interface between the film and the substrate is identified by the yellow arrowheads in the figures. The [010]_pc_ direction of BM-SFO is indicated by a yellow arrow in each HAADF-STEM image. The Sr, Fe, Ti, and Ru cations are displayed by green, red, blue, and yellow spheres, respectively. The data shown here are representative of several SFO/SRO/STO(001) and SFO/STO(001) specimens.

In order to examine the unexpected growth behaviour of BM-SFO thin films in detail, we characterized the interfacial structures of the BM-SFO thin films using scanning transmission electron microscopy (STEM). Atomic-resolution high-angle annular dark-field (HAADF)-STEM images of SFO films were acquired along the [100]_pc,SFO_ direction. These images revealed that SFO thin films grown on SRO/STO(001) and STO(001) had quite different atomic structures near the interfaces, even though they all grew into the BM-SFO phase on the substrates having the same in-plane lattice constants. The images of BM-SFO thin films grown on the SRO/STO(001) substrate showed alternate stacking of bright and dark layers along the *b*-axis at the interface, which corresponded to FeO_6_ octahedra and FeO_4_ tetrahedra in the BM-SFO structure (Fig. 3a). Such a *b*-axis oriented BM-SFO structure appeared over the entire surface of SRO layers. The stacking of octahedral and tetrahedral layers in different orientations, however, was also observed in the BM-SFO film, suggesting that multi-domain growth of the BM-SFO thin film occurred at a few tens-of-nanometres above the interface, possibly due to the strain caused by lattice mismatch between BM-SFO and SRO (see Supplementary Figs. S1 and S2 for the multi-domain structures in BM-SFO and associated functional property of the thin film)^26^.

The atomic arrangement of the BM-SFO thin film at the interface with the SRO layer was investigated using HAADF-STEM imaging and energy dispersive X-ray spectroscopy (EDS) measurements. The Z-contrast nature of HAADF-STEM images clearly revealed the Sr, Fe–O, Ti–O, and Ru–O atomic columns, which are shown as green, red, blue, and yellow spheres in Fig. 3a. The position of the interfaces and atomic columns were accurately verified by EDS element mapping (Fig. 3b). These measurements revealed a remarkably clean interface without misfit dislocation for the BM-SFO films grown on the SRO layer.

In contrast to the film on SRO/STO, the BM-SFO film on the STO substrate showed a quite different interfacial structure. The HAADF-STEM image (Fig. 3c) shows octahedral and tetrahedral layers alternating along the *b*-axis over the entire surface of the STO substrate, indicating *b*-axis growth of the BM-SFO thin film; this is in accord with the XRD measurements (Fig. 2c). The *b*-axis oriented growth of the BM-SFO thin film is identical to that of BM-SFO grown on SRO (Fig. 3a). However, multi-domain growth of BM-SFO, which was observed for BM-SFO/SRO/STO(001), was not observed within the thin film. Additionally, a dark stripe, identified by the green-dashed rectangle in Fig. 3c, was observed at the position where the alternate stacking of bright and dark layers began to form along the *b*-axis. This stripe, which corresponds to a planar defect, formed horizontally and was present even within the films (other types of interfacial structures observed between BM-SFO and STO are shown in Supplementary Fig. S3).

Interestingly, the HAADF-STEM imaging and EDS measurements (Fig. 3c,d) clearly revealed a transition layer between the BM-SFO thin film and the STO substrate. The position of the dark stripe marked by the green-dashed rectangle in Fig. 3c was not located at the interface between the BM-SFO thin film and the STO substrate. The crystal structure of the BM-SFO film on the STO substrate maintained a *cubic perovskite-like* structure, the same as that of the underlying STO substrate, and changed into the brownmillerite structure mostly with the formation of planar defects within the range of 1–5 nm above the BM-SFO/STO(001) interface. Such planar defects corresponded to Ruddlesden–Popper (RP) planar faults that consisted of a double atomic layers of Fe-O rock-salt structure formed on the surface layer of the *perovskite-like* SFO with a shear shift of 1/2[110]^27,28^.

**Figure 4:**
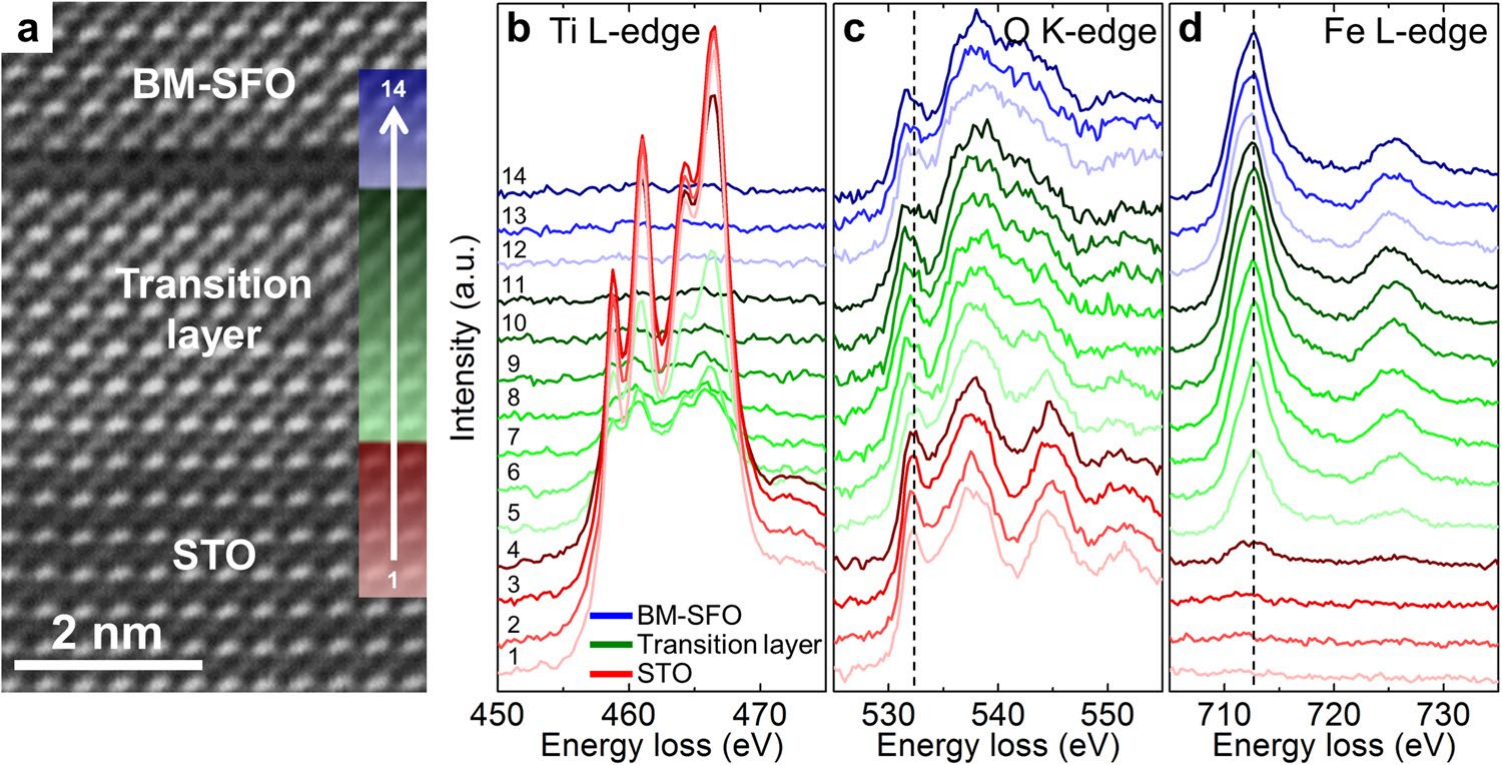
Identification of the transition layer using EELS measurements. **(a)** HAADF-STEM image of BM-SFO/STO(001) at the interface. **(b–d)** A series of ELNES at the (**b**) Ti *L*_2,3_-edge, (**c**) O *K*-edge, and (**d**) Fe *L*_2,3_-edge. These spectra were acquired across the heterointerface from the STO substrate through the transition layer to the BM-SFO thin film and correspond to the regions coloured red, green, and blue, respectively, in. (**a**) Each spectrum is labelled in order from “1” (STO) to “14” (BM-SFO). The black dashed lines in (**c**,**d**) represent the energy values of the O *K*-edge pre-peak for the STO substrate and the Fe *L*_3_-edge for the BM-SFO thin film, respectively.

Electron energy loss spectrum (EELS) measurements were conducted to identify the transition layer having the unusual *perovskite-like* SFO structure. Figure 4a shows a HAADF-STEM image of BM-SFO/STO(001) at the interface. The electron-loss near-edge structures (ELNES) of the Ti and Fe *L*_2,3_-edges and the O *K*-edge were measured across the interface from the STO substrate through the transition layer to the BM-SFO thin film (Fig. 4b–d). The EELS spectra of the STO substrate and the BM-SFO thin film exhibited characteristic Ti (red in Fig. 4b) and Fe *L*_2,3_-edge (blue in Fig. 4d) structures such as spin-orbit and crystal field splitting, which are identical to those of the stoichiometric compounds^29,30^. The intensity of the Ti *L*_2,3_-edge gradually decreased from the STO substrate to the BM-SFO thin film across the transition layer, while that of the Fe *L*_2,3_-edge increasingly increased. The presence of both Ti and Fe *L*_2,3_-edges at the boundary between the STO substrate and the transition layer indicated slight cation intermixing between Ti and Fe, *i.e*. SrTi_(1−x)_Fe_x_O_y_, within a few atomic layers. The intensity of the Ti *L*_2,3_-edge decayed to zero above the intermixing layer, while that of Fe *L*_2,3_-edge reached nearly its maximum value within the transition layer, indicating that the transition layer above the intermixing layer consisted of the SFO phase. The thickness of the *perovskite-like* SFO layer varied slightly with position (see Supplementary Fig. S4 for more details).

The valence state of Fe in the *perovskite-like* SFO phase in the transition layer was further investigated using EELS analysis. The peaks corresponding to the Ti and Fe *L*_2,3_-edges remained at exactly the same energy loss across the transition layer (Spectra 1–14, Fig. 4b,d), which suggests that the chemical shifts for the Ti and Fe core levels were negligible within the transition layer. Importantly, this indicates that the valence state of Fe was still 3+, the same as in the BM-SFO phase, throughout the transition layer despite its *perovskite-like* crystal structure verified by HAADF-STEM images.

The O *K*-edge ELNES of the *perovskite-like* SFO layer were also similar to those of the BM-SFO thin film. The STO substrate and the BM-SFO thin film showed their characteristic O K-edge structures, as displayed in red and blue in Fig. 4c, respectively^29,30^. The O *K*-edge spectrum from the STO substrate consists of the first pre-peak and second and third main peaks, in ascending order of energy loss; these are derived from the O 2*p* state hybridized with Ti 3*d*, Sr 4*d*, and Ti 4*sp* states, respectively^31^. The pre-peak shifted toward lower energy loss, and other two peaks broadened and merged together from the STO substrate to the *perovskite-like* SFO layer. The O *K*-edge spectra then remained nearly unchanged throughout the transition layer and became similar to that of the BM-SFO phase showing the Fe oxidation state of 3+ (oxygen concentration across the interface and the differences in the EELS spectra between SrFeO_3_ and SrFeO_2.5_ can be found in Supplementary Fig. S5 and Supplementary Information S6). The only difference was that the pre-peak was blunt at the RP planar faults; this was attributed to the different electronic structures of the RP and BM-SFO phases.

**Figure 5:**
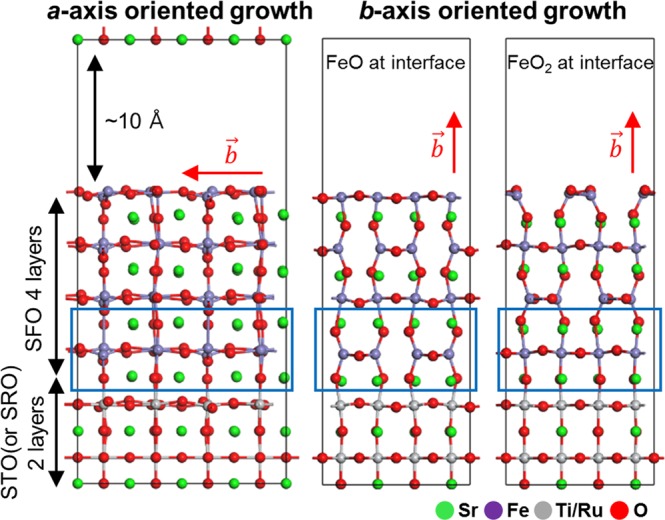
First principles simulations of atomic structures at the BM-SFO/STO(001) and BM-SFO/SRO(001) interfaces. Four layers of BM-SFO with growth directions along the *a*- and *b*-axes were placed on two layers of STO (or SRO). For *b*-axis growth, two different interfacial structures having FeO and FeO_2_ layers at the interface were calculated. The Sr, Fe, Ti (Ru) cations, and O atoms are displayed by green, violet, grey, and red spheres, respectively. The atomic configurations of BM-SFO at the interfaces are highlighted by blue rectangles.

These STEM measurements clearly revealed that BM-SFO thin films were grown along *b*-axis on the clean interface of SRO/STO(001) and on STO(001) together with the formation of the transition layer at the heterointerface. Such *b*-axis oriented growth of the BM-SFO thin films on both STO(001) and SRO/STO(001) substrates was opposite to the preferential *a*-axis oriented growth expected on the basis of epitaxial strain energy considering simple lattice mismatches. To account for the discrepancy between the experimental results and the prediction from the conventional epitaxial strain effect, first principles calculations were performed. To identify the energetically favourable interfacial structure, we built atomistic interface models taking into account two possible orientations of BM-SFO, *i.e*., along the *a*- and *b*-axes (Fig. 5), and compared their heats of formation according to the equation:

1 $$\Delta {H}_{f}=\frac{1}{A}\{{E}_{{\rm{slab}}}-m{E}_{{\rm{substrate}}}-n{E}_{{{\rm{SrFeO}}}_{2.5}}\}$$

where *A* is the in-plane area of the interface model, *E*_*slab*_ is its energy including the contribution of the surfaces as well as the interfaces, *E*_substrate_ is the energy of the STO or SRO unit cell, ${E}_{{{\rm{SrFeO}}}_{2.5}}$ is the energy of the SrFeO_2.5_ unit cell, and *m* and *n* denote the number of formula units of the substrate and SrFeO_2.5_ included in the interface model, respectively. Because FeO and FeO_2_ layers are alternately stacked along the *b*-axis in BM-SFO, for the *b*-axis orientation of BM-SFO we examined these two structures with the different formula units, namely FeO or FeO_2_, at the interface. In our interface models, four layers of SFO lay on top of two layers of STO (or SRO) and their atomic positions were fully relaxed except for the one layer of STO (or SRO) at the bottom, which was considered to be bulk. The in-plane lattice parameters of supercells were fixed to those of STO in every calculation, which reflected the coherent growth of SRO as well as SFO in experiments.

The heats of formation in Eq. (1) indicate the contribution of the surfaces as well as the interface of the atomistic interface models. Both surface and interface energies are crucial to determine the growth behaviour of thin film at the initial growth stage. Thus, the calculations based on our interface models are suitable to compare the stability of each system relatively for the growth of BM-SFO thin films. Table 1 lists the Δ*H*_*f*_ for SFO/SRO/STO(001) and SFO/STO(001).

**Table 1:** Heats of formation per unit volume at the interface of BM-SFO/SRO(001) and BM-SFO/STO(001). The calculated energies were obtained following the complete structural optimization represented in Fig. 5.

		*a*-axis	*b*-axis	
			FeO	FeO_2_
**Δ*H***_***f***_ [meV Å^−3^]	**SrTiO**_**3**_**/SrFeO**_**2.5**_	144	220	176
	**SrRuO**_**3**_**/SrFeO**_**2.5**_	133	156	112

Interestingly, BM-SFO preferred *b*-axis growth with FeO_2_ units at the interface of the SRO/STO(001) substrate, despite its significant in-plane area mismatch. The reasons for this unexpected result are twofold. First, different chemical elements in the B-site of a BO_6_ octahedron result in different interfacial effects for the growth of BM-SFO thin films. The RuO_6_ octahedra in SRO and FeO_6_ ones in SFO are all slightly rotated and tilted in their bulk forms and can be connected coherently to each other through the formation of FeO_6_ octahedral layers at the interface^16,32^. Such bond coherency between distorted RuO_6_ and FeO_6_ octahedra may favour the *b*-axis growth of BM-SFO, which improves the *octahedral connectivity* at the interface compared with the formation of alternating FeO_6_ octahedral and FeO_4_ tetrahedral layers along the surface of the SRO/STO(001) substrate. Therefore, *b*-axis oriented growth of a BM-SFO thin film is further stabilized on the SRO/STO(001) substrate. Second, according to previous studies, oxygen is more strongly bound in SRO than in SFO, which makes the oxygen-rich unit, *i.e*., FeO_2_, more favourable at the interface than FeO^33^.

Indeed, the interfacial *octahedral connectivity* plays a significant role in altering the oxygen coordination environment of a thin film at the heterointerface. The corner-connectivity of BO_6_ octahedra allows an oxide film to mimic the octahedral rotation and tilt, which are transferred across the heteroepitaxial interface from the substrate^34–36^. These changes in the octahedral framework provide new crystal symmetry and physical properties to the films, such as magnetic and transport properties, which are inaccessible in their bulk phases^12–14,37^. Meanwhile, we found that the *octahedral connectivity* functioned as a new type of constraint influencing the growth direction of a thin film having an anisotropic structure. The first deposition layer was a pure FeO_6_ octahedral layer, which provided better octahedral framework coupling between the film and the substrate and consequently led to the stacking of octahedral and tetrahedral layers in a vertical direction with respect to the substrate.

In contrast to the SRO/STO substrates, calculation results on the STO substrates contradict to the experimental results; *a*-axis growth of BM-SFO was calculated to be most stable on the STO(001) substrate, opposed to the experimentally observed *b*-axis growth in Figs. 2c and 3c. Such a discrepancy is possibly attributed to the transition layer observed at the heterointerface of BM-SFO/STO(001), which was not considered for the calculations. The ideal interface model exhibiting an abrupt interface cannot be directly applicable to predict the growth behaviour of BM-SFO thin film on the STO(001) substrate, whereas this model is appropriate for the calculations of BM-SFO/SRO(001) structure with a clean interface (Fig. 3a).

SFO in the transition layer had a *perovskite-like* crystal structure and an unusual Fe valence state of 3+, as revealed by HAADF-STEM and EELS measurements. The cubic SrFeO_2.5_ phase with a lattice constant of 3.970 Å and Fe oxidation state of 3+ normally appears above 830 °C by disturbing ordered oxygen vacancy channels^38^. However, the cubic SrFeO_2.5_ phase with disordered oxygen vacancies was also stabilized at the interface between BM-SFO and DyScO_3_ by accommodating the in-plane area mismatch without elevating the temperature^39^. For the BM-SFO/STO(001) heterointerface in the present study, such disordered SFO phase and RP planar defects just above the interface may act as a buffer layer, thereby stabilizing *b*-axis oriented growth of BM-SFO without forming the multi-domain structure and dislocations by relieving the epitaxial strain; this was not considered in the first principles calculations of Fig. 5. This *b*-axis oriented growth might be stabilized by lower lattice mismatch between *perovskite-like* SFO and BM-SFO. The in-plane area mismatch of *perovskite-like* SFO (3.970 × 3.970 Å^2^) with the *ac*-plane of BM-SFO (4.010 × 3.910 Å^2^) is lower than with the *bc*-plane of BM-SFO (3.900 × 3.910 Å^2^), which energetically leads to *b*-axis oriented growth of the BM-SFO thin film. The interfacial *octahedral connectivity* considered for BM-SFO/SRO/STO(001) cannot be applied in this case due to the disturbed octahedral framework of *perovskite-like* SFO. The metastable RP faults are normally generated during a thermodynamically nonequilibrium thin film growth process at low temperature, depending on the growth condition used in pulsed laser deposition (PLD) and molecular beam epitaxy^27^. Therefore, the initial growth condition in the present study might have induced formation of the RP phase and the *perovskite-like* SFO layer with disordered oxygen vacancies. The influence of such a buffer layer on the growth behaviour of the thin films signifies interfacial effects induced by a specific growth condition.

## Conclusion

In conclusion, we demonstrated that the growth process of a BM-SFO thin film can be determined through novel interfacial effects that overwhelm the conventional lattice mismatch argument. These interfacial effects seem to originate from the *peculiar transition layer* as well as *octahedral connectivity* and chemical composition at the interface. The BM-SFO thin films grown on two types of substrates, *i.e*., STO(001) and SRO/STO(001), exhibited *b*-axis oriented growth with distinct microstructures and interfacial structures, whereas *a*-axis oriented growth was predicted for both substrates by the simple epitaxial strain effect. Bond coherency between distorted octahedra of RuO_6_ and FeO_6_ along the *b*-axis presumably stabilized *b*-axis growth of BM-SFO on the SRO/STO(001) substrate, which mimicked the atomic configuration of the SRO buffer layer at the interface. Our concept of *octahedral connectivity* could be one of the missing links between conventional strain engineering based on simple lattice mismatch and the concept of *octahedral-tilt connectivity*. Meanwhile, the *cubic perovskite-like* SrFeO_2.5_ phase with disordered oxygen vacancies and RP phase were self-generated and served as a buffer layer, resulting in *b*-axis oriented growth of the BM-SFO on the STO(001) substrate. Even these two phases can be roughly understood in terms of *octahedral connectivity*.

Understanding the growth mechanisms is an essential prerequisite for the manipulation and fabrication of desired oxide heterostructures. Here, we clearly revealed two different growth mechanisms governing the same b-axis oriented growth behavior for BM-SFO/STO(001) and BM-SFO/SRO/STO(001). These findings greatly enhance our understanding of the parameters affecting heterointerfacial structures, which determine the crystal growth behaviour of complex oxide thin films such as BM oxides. More importantly, our results are expected to stimulate research on interfacial effects that could be used to tune the growth process of oxide thin films and the interfacial structure. Controlling the growth process, *e.g*., the direction of oxygen vacancy channels, by tailoring the heterointerface could lead to potentially new device applications such as solid-oxide fuel cells and resistive-switching memory.

## Methods

### BM-SFO thin film growth

Epitaxial SFO and SRO thin films were grown on the STO(001) substrate using PLD with a KrF excimer laser and commercial (Toshima, Japan) and homemade targets. For the thin film growth, STO substrate was etched using a buffered NH_4_F-HF (BHF) solution with pH = 4.5. The STO substrate was immersed in the BHF solution for 30 s, rinsed with pure water and ethanol, dried in a nitrogen stream, and then annealed at 1050 °C to obtain a well-defined step-and-terrace STO surface. The 60–70-nm-thick SFO layer was deposited at a substrate temperature of 650 °C, oxygen partial pressure of 10 mTorr, laser fluence of *ca*. 2.1 J cm^−2^, and a repetition rate of 4 Hz. To prepare the BM-SFO/SRO/STO heterostructure, a 60–70-nm-thick SRO buffer layer was first deposited (substrate temperature: 750 °C; repetition rate: 4 Hz; fluence: *ca*. 2.5 J cm^−2^) and then the 60–70-nm-thick SFO layer was deposited on top of the SRO layer. Details of the thin film fabrication are reported elsewhere^23,25^.

### First principles calculations

First principles calculations were carried out using the Vienna *ab initio* Simulation Package^40^. We used the projector-augmented wave method to describe electron-ion interactions^41^. The PBE-GGA functional was used to calculate the exchange-correlation energy^42^ and an effective on-site energy, *U*_eff_, of 4.3 eV for the Fe 3*d* states. The cutoff energy for the plane-wave basis set was set to 550 eV. The 1 × 3 × 1 and 4 × 4 × 1 k-point meshes were used to model the *a*-axis and *b*-axis growth of the BM-SFO thin films, respectively. Spin-polarized calculations were conducted for all models. The spurious dipole interaction between the slabs was corrected by introducing external dipoles^43^.

### Structural and microstructural characterization

The epitaxial structures of the BM-SFO/SRO/STO(001) and BM-SFO/STO(001) samples were investigated using a high-resolution X-ray diffractometer (Bruker D8). Microstructural analysis of the interfacial structures was performed by TEM. Cross-sectional TEM specimens were prepared by focused ion beam (FIB) milling (Helios 650 FIB, FEI) and thinned by focused Ar-ion milling (NanoMill 1040, Fischione). The atomic-resolution HAADF-STEM images and EDS maps were acquired using a spherical aberration-corrected TEM (JEM-ARM200F, JEOL) equipped with a cold field emission gun. Spatially resolved EELS measurements were performed by spectral mapping with a spherical aberration-corrected TEM (Titan Themis 60–300 cubed, FEI) equipped with double *Cs* correctors, a high-brightness X-FEG module with monochromator, and a GATAN GIF Quantum ERS/966 energy filter. The spectra were recorded using a 300-kV monochromated electron beam. The spectra obtained along the in-plane direction were summed to improve the signal-to-noise ratio. The background was subtracted using the power law. The convergence semi-angle of the monochromated probe was 28.2 mrad, and the acceptance semi-angle for EELS was 40.7 mrad.

## Supplementary information

Supplementary information.

## Data Availability

The datasets generated during and/or analysed during the current study are available from the corresponding author on reasonable request.

## References

[1] Goodenough JB (2004). Electronic and ionic transport properties and other physical aspects of perovskites. Rep. Prog. Phys..

[2] Zubko P, Gariglio S, Gabay M, Ghosez P, Triscone JM (2011). Interface Physics in Complex Oxide Heterostructures. Annu. Rev. Conden. Ma. P..

[3] Ohtomo A, Hwang HY (2004). A high-mobility electron gas at the LaAlO_3_/SrTiO_3_ heterointerface. Nature.

[4] Nakagawa N, Hwang HY, Muller DA (2006). Why some interfaces cannot be sharp. Nat. Mater..

[5] Reyren N (2007). Superconducting interfaces between insulating oxides. Science.

[6] Mannhart J, Schlom DG (2010). Oxide Interfaces-An Opportunity for Electronics. Science.

[7] Tsukazaki A (2010). Observation of the fractional quantum Hall effect in an oxide. Nat. Mater..

[8] Ohtomo A, Muller DA, Grazul JL, Hwang HY (2002). Artificial charge-modulation in atomic-scale perovskite titanate superlattices. Nature.

[9] Ogawa N, Satoh T, Ogimoto Y, Miyano K (2008). Nonlinear optical detection of a ferromagnetic state at the single interface of an antiferromagnetic LaMnO3/SrMnO3 double layer. Phys. Rev. B.

[10] Gozar A (2008). High-temperature interface superconductivity between metallic and insulating copper oxides. Nature.

[11] Izumi M (2001). Perovskite superlattices as tailored materials of correlated electrons. Mat. Sci. Eng. B-Solid.

[12] Kan D (2016). Tuning magnetic anisotropy by interfacially engineering the oxygen coordination environment in a transition metal oxide. Nat. Mater..

[13] Liao Z (2016). Controlled lateral anisotropy in correlated manganite heterostructures by interface-engineered oxygen octahedral coupling. Nat. Mater..

[14] O’Sullivan M (2016). Interface control by chemical and dimensional matching in an oxide heterostructure. Nat. Chem..

[15] Jeen H (2013). Topotactic Phase Transformation of the Brownmillerite SrCoO_2.5_ to the Perovskite SrCoO_3−δ_. Adv. Mater..

[16] Young J, Rondinelli JM (2015). Crystal structure and electronic properties of bulk and thin film brownmillerite oxides. Phys. Rev. B.

[17] Boivin JC, Mairesse G (1998). Recent material developments in fast oxide ion conductors. Chem. Mater..

[18] Orera A, Slater PR (2010). New Chemical Systems for Solid Oxide Fuel Cells. Chem. Mater..

[19] Tambunan OT (2014). Resistance switching in epitaxial SrCoO_x_ thin films. Appl. Phys. Lett..

[20] Inoue S (2010). Anisotropic oxygen diffusion at low temperature in perovskite-structure iron oxides. Nat. Chem..

[21] Auckett JE (2013). Combined Experimental and Computational Study of Oxide Ion Conduction Dynamics in Sr_2_Fe_2_O_5_ Brownmillerite. Chem. Mater..

[22] Mitra C, Meyer T, Lee HN, Reboredo FA (2014). Oxygen diffusion pathways in brownmillerite SrCoO_2.5_: Influence of structure and chemical potential. J. Chem. Phys..

[23] Acharya SK (2017). Brownmillerite thin films as fast ion conductors for ultimate-performance resistance switching memory. Nanoscale.

[24] Rossell MD (2004). Structure of epitaxial Ca_2_Fe_2_O_5_ films deposited on different perovskite-type substrates. J. Appl. Phys..

[25] Acharya SK (2016). Epitaxial Brownmillerite Oxide Thin Films for Reliable Switching Memory. Acs Appl. Mater. Inter..

[26] Shimakawa Y (2010). Topotactic Changes in Thin Films of Brownmillerite SrFeO_2.5_ Grown on SrTiO_3_ Substrates to Infinite-Layer Structure SrFeO_2_. Cryst. Growth Des..

[27] Suzuki T, Nishi Y, Fujimoto M (2000). Ruddlesden-Popper planar faults and nanotwins in heteroepitaxial nonstoichiometric barium titanate thin films. J. Am. Ceram. Soc..

[28] Klie RF, Browning ND (2002). Atomic scale characterization of vacancy ordering in oxygen conducting membranes. Microsc. Microanal..

[29] Galakhov VR (2010). Valence Band Structure and X-ray Spectra of Oxygen-Deficient Ferrites SrFeO_x_. J. Phys. Chem. C.

[30] Zhu GZ, Radtke G, Botton GA (2012). Bonding and structure of a reconstructed (001) surface of SrTiO_3_ from TEM. Nature.

[31] Seo H (2012). Band alignment and electronic structure of the anatase TiO_2_/SrTiO_3_(001) heterostructure integrated on Si(001). Phys. Rev. B..

[32] Haruta M (2011). Local electronic structure analysis for brownmillerite Ca(Sr)FeO_2.5_ using site-resolved energy-loss near-edge structures. J. Appl. Phys..

[33] Berger RF, Broberg DP, Neaton JB (2014). Tuning the electronic structure of SrTiO_3_/SrFeO_3−x_ superlattices via composition and vacancy control. Apl. Mater..

[34] Rondinelli JM, May SJ, Freeland JW (2012). Control of octahedral connectivity in perovskite oxide heterostructures: An emerging route to multifunctional materials discovery. Mrs Bull..

[35] Aso R, Kan D, Shimakawa Y, Kurata H (2013). Atomic level observation of octahedral distortions at the perovskite oxide heterointerface. Sci. Rep..

[36] Kan D, Aso R, Kurata H, Shimakawa Y (2015). Phase control of a perovskite transition-metal oxide through oxygen displacement at the heterointerface. Dalton T..

[37] He J, Borisevich A, Kalinin SV, Pennycook SJ, Pantelides ST (2010). Control of Octahedral Tilts and Magnetic Properties of Perovskite Oxide Heterostructures by Substrate Symmetry. Phys. Rev. Lett..

[38] Grenier JC, Ea N, Pouchard M, Hagenmuller P (1985). Structural Transitions at High-Temperature in Sr_2_Fe_2_O_5_. J. Solid State Chem..

[39] Hirai K (2017). Melting of Oxygen Vacancy Order at Oxide-Heterostructure Interface. Acs Appl. Mater. Inter..

[40] Kresse G, Furthmuller J (1996). Efficient iterative schemes for ab initio total-energy calculations using a plane-wave basis set. Phys. Rev. B.

[41] Blochl PE (1994). Projector Augmented-Wave Method. Phys. Rev. B.

[42] Perdew JP, Burke K, Ernzerhof M (1996). Generalized gradient approximation made simple. Phys. Rev. Lett..

[43] Bengtsson L (1999). Dipole correction for surface supercell calculations. Phys. Rev. B.

