# Supplementary material for: Effects of the Heterointerface on the Growth Characteristics of a Brownmillerite SrFeO2.5 Thin Film Grown on SrRuO3 and SrTiO3 Perovskites
Source: Sci Rep. 2020 Mar 2;10:3807. doi: 10.1038/s41598-020-60772-2 (PMC7052257; doi:10.1038/s41598-020-60772-2)
Supplement: Supplementary file 1 — Supplementary information. [file 41598_2020_60772_MOESM1_ESM.docx]

Supplementary Information

Effects of the Heterointerface on the Growth Characteristics of a Brownmillerite SrFeO_2.5_ Thin Film Grown on SrRuO_3_ and SrTiO_3_ Perovskites

Janghyun Jo^1^, Venkata Raveendra Nallagatlla^2^, Susant Kumar Acharya^2^, Youngho Kang^1^, Yoonkoo Kim^1^, Sangmoon Yoon^1^, Sangmin Lee^1^, Hionsuck Baik^3^, Seungwu Han^1^, Miyoung Kim^1,*^ and Chang Uk Jung^2,*^

^1^Department of Materials Science and Engineering and Research Institute of Advanced Materials, Seoul National University, Seoul 08826, Republic of Korea
* E-mail: mkim@snu.ac.kr
^2^Department of Physics and Oxide Research Centre, Hankuk University of Foreign Studies, Yongin 17035, Republic of Korea

* E-mail: cu-jung@hufs.ac.kr

^3^Seoul Center, Korea Basic Science Institute, Seoul 136-713, Republic of Korea

**S1. Multi-domain structure of BM-SFO thin film**


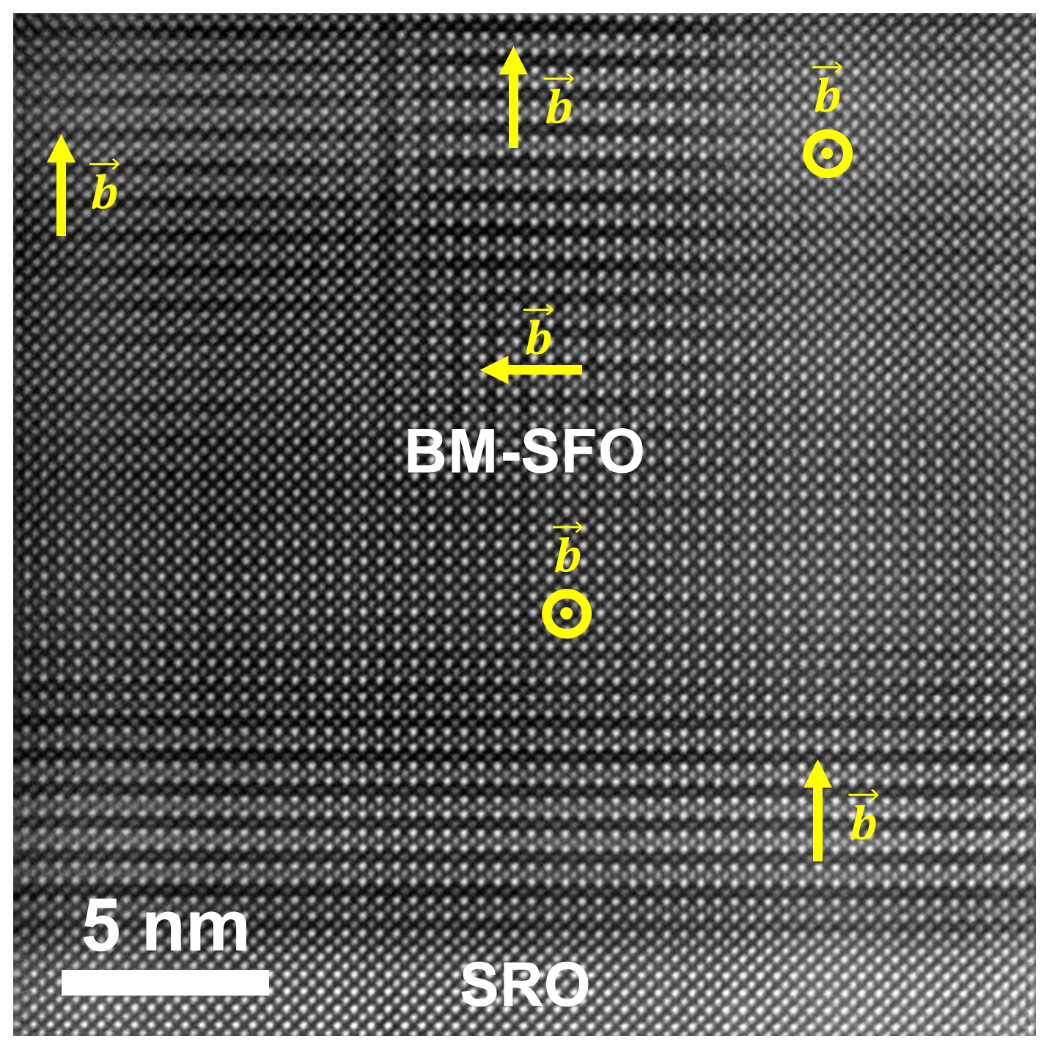


**Figure S1.** Cross-sectional HAADF-STEM image showing a multi-domain structure of BM-SFO thin film grown on SRO/STO(001). Homogeneous region showing the stacking of FeO_6_ octahedra and FeO_4_ tetrahedra along the *b*-axis is observed right above the SFO/SRO heterointerface, as also shown in Fig. 3a. BM-SFO domains with three orthogonal orientations, on the other hand, are observed over the homogeneous region. This indicates the following growth mechanisms; single crystalline BM-SFO thin film is grown along *b*-axis on the whole surface of SRO/STO(001) substrate, then BM-SFO multi-domains form with three different growth orientations of [100]_pc_, [010]_pc_, and [001]_pc_ to relax the strain from the lattice mismatch with the substrate. The preferential growth direction of the BM-SFO thin film in the multi-domain region was found to be along the *b*-axis. The direction of the *b*-axis of the BM-SFO phase is identified with yellow arrows in the domains.

**S2. Nonuniform resistive switching behavior of Au/BM-SFO/SRO/STO device**

**
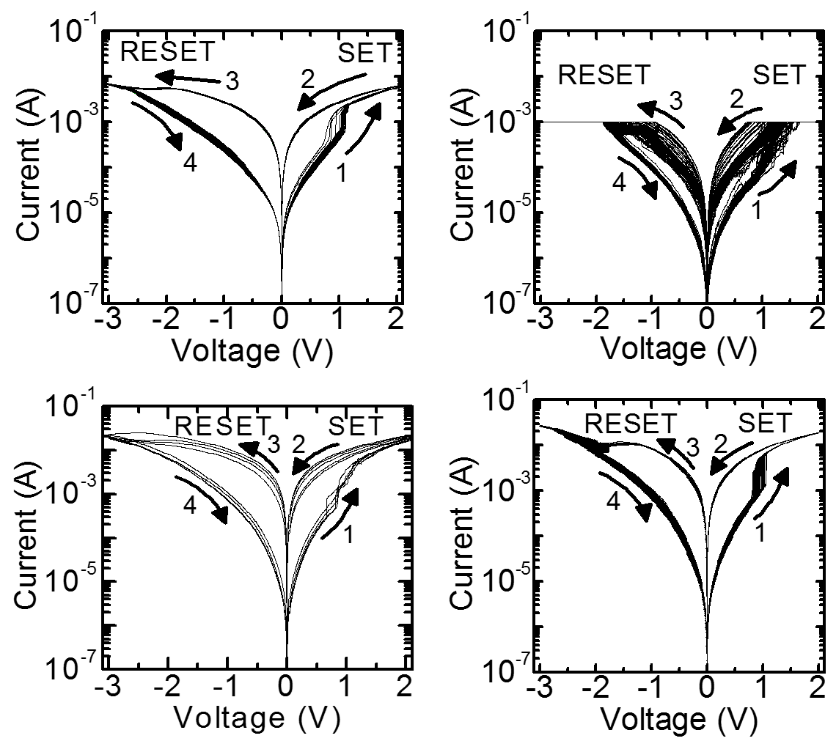
**

**Figure S2.** I-V hysteresis loops in four different Au/BM-SFO/SRO resistive switching devices.

Structural inhomogeneity such as multi-domain structure formed within BM-SFO thin film might cause unreliable resistive switching behaviour of the device. BM-SFO/SRO/STO(001) system was applied to the resistive switching device with Au and SRO as top (TE) and bottom electrodes (BE), respectively. Figure S2 shows four I-V curves during resistive switching of four different Au(TE)/BM-SFO/SRO(BE) devices with multi-domain structure in BM-SFO film. These four I-V hysteresis display completely different resistive switching behaviour, such as SET and RESET voltages, and resistances in high and low resistance states, representing poor device to device uniformity. Nonuniform resistive switching behaviour during repeated cycles was also observed even in a single device. Such a nonuniform switching behaviour might be associated with multi-domain structures in the BM-SFO film which hinder the motion of oxygen vacancies in the film, and thus the formation of conducting filaments between both electrodes.^1,2^ From these results, we may conclude that controlling and understanding the structural homogeneity of a switching layer during crystal growth have significant implications in realizing the uniform and reliable resistive switching behaviour.

**S3. Other types of heterointerface between the BM-SFO thin film and the STO substrate**


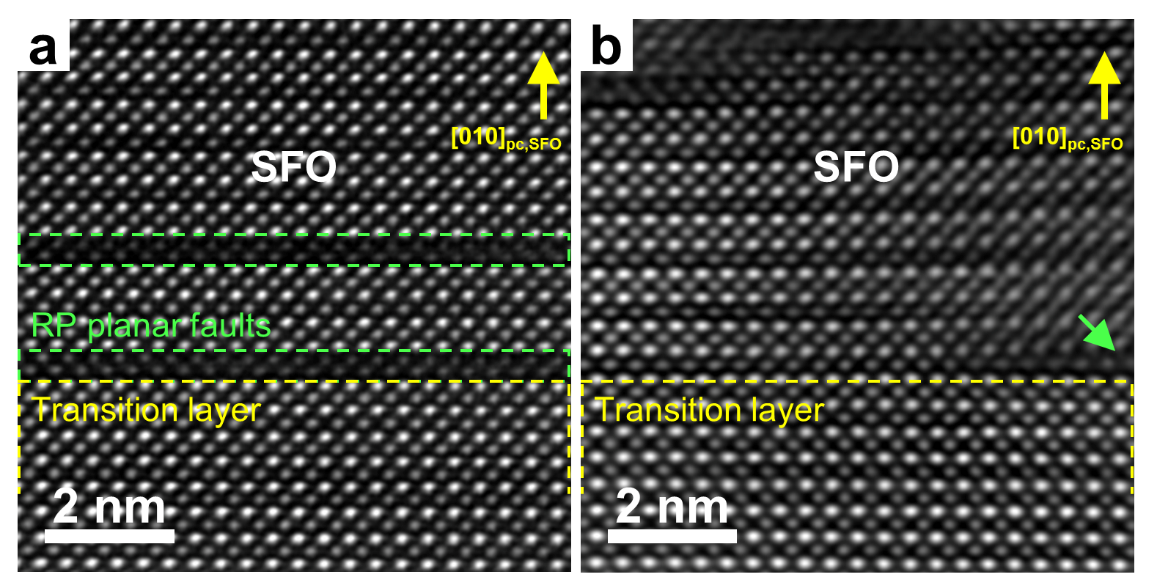


**Figure S3.** Interfacial structures between BM-SFO and the STO substrate. **(a)** Double RP planar defects formed above the transition layer at the heterointerface. **(b)** No RP planar defects at the heterointerface. A planar defect began to appear at the position marked with a green arrow. The heterointerface was mainly composed of a transition layer and an RP planar defect (Fig. 3c), but other types of interface were also present, as shown in (a) and (b).

**S4. Variation in the thickness of the *perovskite-like* SFO layer**


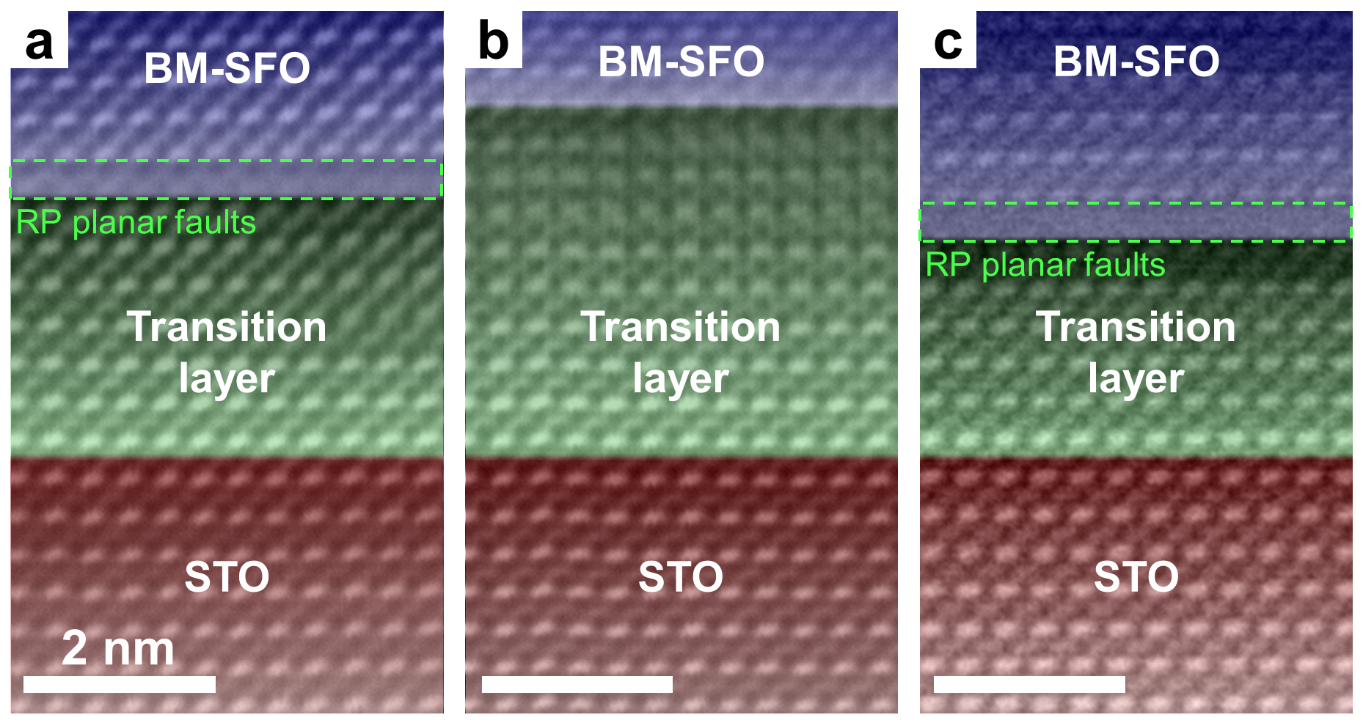


**Figure S4.** Thickness variation of the *perovskite-like* SFO layer. **(a–c)** HAADF-STEM images of the BM-SFO/STO(001) heterointerface at three different locations. The regions corresponding to the STO substrate, transition layer, and BM-SFO thin film are coloured in red, green, and blue, respectively. These regions were verified by a series of EELS measurements across the heterointerface; the same procedures were performed in Fig. 4. The thickness of the *perovskite-like* SFO layer varied slightly with location, ranging from 1–5 nm.

**S5. Oxygen concentration across the thin film/transition layer interface**


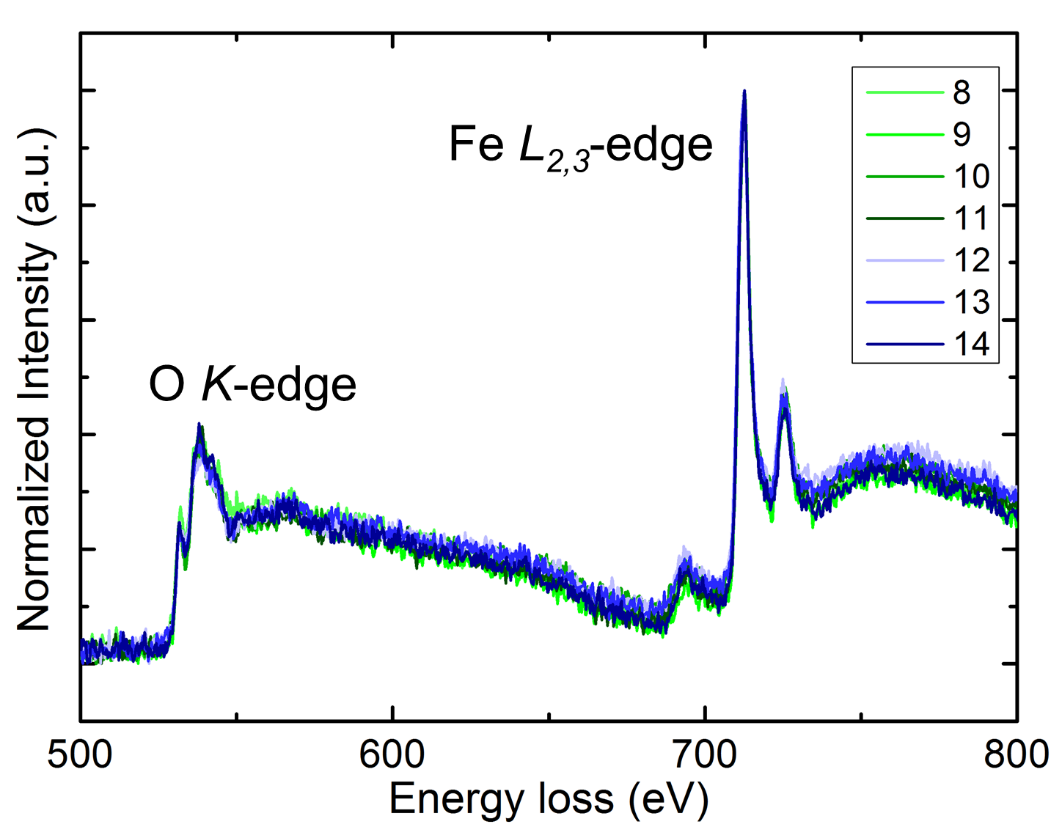


**Figure S5.** A series of ELNES acquired across the interface between the transition layer and the BM-SFO thin film of the BM-SFO/STO(001) specimen. For the comparison of oxygen concentration, the spectra are normalized by the maximum intensity of Fe *L*_3_-edge peaks and overlapped each other. The graph clearly shows that the intensities of the O *K*-edge peaks and their backgrounds are almost same, which indicates that the oxygen concentration almost remain constant across the interface. This further confirms that the transition layer exhibits the SrFeO_2.5_ phase and thus the Fe valence state of 3+. Each spectrum is labelled in the same manner as Fig. 4 (8–11: transition layer, 12–14: BM-SFO thin film).

**S6. Discussion on the EELS spectra in SrFeO_3_ and SrFeO_2.5_**

The cubic structure in the transition layer may originates from the SrFeO_3_ phase having an oxygen stoichiometry of 3. If so, the valence state of Fe would be 4+, which exhibit different Fe *L*-edge from that of BM-SFO phase having the Fe valence state of 3+. The valence state of SrFeO_3-x_ can be investigated by ELNES and X-ray absorption near edge structure that exhibit different features, *i.e.* chemical shift or fine structure shape, depending on the valence state of the element in the material. Q. Liu *et al.* reported that the peak position in the Fe *L*_2,3_-edge of SrFeO_2.5_ is 1–2 eV lower than that of SrFeO_3_.^3^ Additionally, M. Haruta *et al.* and J.-S. Kang *et al.* reported that SrFeO_2.5_ and SrFeO_3_ exhibit different O *K*-edge shapes.^4,5^ These results clearly illustrate differences in the near-edge structures depending on the valence state of Fe and oxygen stoichiometry of the compound. However, this is not the case in our EELS results as shown in Fig. 4, which suggests the same valence state and oxygen stoichiometry between the transition layer and the BM-SFO thin film.

**References**

1 Acharya, S. K. *et al.* Epitaxial Brownmillerite Oxide Thin Films for Reliable Switching Memory. *Acs Appl. Mater. Inter.* **8,** 7902-7911 (2016).

2 Acharya, S. K. *et al.* Brownmillerite thin films as fast ion conductors for ultimate-performance resistance switching memory. *Nanoscale* **9,** 10502-10510 (2017).

3. Liu, Q., *doctoral dissertation*, University of Bordeaux 1 (Bordeaux, France) (2013**)**.

4. Kang, J. S. *et al.* *Phys. Rev. B* **78,** 5 (2008).

5 Haruta, M. *et al.* Local electronic structure analysis for brownmillerite Ca(Sr)FeO_2.5_ using site-resolved energy-loss near-edge structures. *J. Appl. Phys.* **110,** 8 (2011).
